# Role of TRP Channels in Cancer-Induced Bone Pain

**DOI:** 10.3390/ijms26031229

**Published:** 2025-01-30

**Authors:** Flaminia Coluzzi, Maria Sole Scerpa, Elisa Alessandri, Patrizia Romualdi, Monica Rocco

**Affiliations:** 1 Department of Surgical and Medical Sciences and Translational Medicine, Sapienza University of Rome, 00189 Rome, Italy; 2 Unit Anesthesia, Intensive Care and Pain Therapy, Sant’Andrea University Hospital, 00189 Rome, Italy; 3 Department of Pharmacy and Biotechnology, Alma Mater Studiorum—University of Bologna, 40126 Bologna, Italy

**Keywords:** TRP channels, TRPV1, TRPA1, TRPM8, opioids, bone, cancer pain, metastasis, resiniferatoxin, neuropathic pain

## Abstract

The burden of cancer is growing in almost every country. Bone metastases significantly affect the prognosis and lead to an increase in mortality and morbidity. The management of cancer-induced bone pain (CIBP) still shows various unmet needs. Opioid use is burdened by a number of possible side effects. Moreover, recent progresses in cancer treatment significantly increased the life expectancy of cancer patients, even those with metastatic disease. In this narrative review, we reported the main findings regarding TRP channel function in cancer pain models. TRP cation channels play a key role in different functions of cancer cells, including the regulation of their potential for metastasization, and are the main channels involved in the pathways of pain perception, through peripheral and central effects. Genetic deletion decreased pain sensitivity following tumour cell inoculation. Preclinical data suggest a potential role for modulators of some TRP channels, such as TRPV1, TRPA1, TRPM7 and TRPM8. Clinical results are still scarce; however, the physiological role in modulating bone remodelling and the involvement of TRP channels in preclinical models of bone cancer pain have garnered interest as areas of research in the last few years, as innovative analgesic strategies that may overcome the long-term side effects of opioids.

## 1. Introduction

The burden of cancer is growing in almost every country, reaching a worldwide total of about 20 million new cases diagnosed in 2022 [1]. Bone is one of the most common targets of metastatic disease, with the highest prevalence of bone metastases observed in the lung (44.4%), prostate (19.3%), breast (12.3%), kidney (4.0%), colorectal area (2.24%), and pancreas (2.23%) [2]. Bone metastases can represent an onset symptom of the oncological disease. De novo bone metastases, detected at the time of primary cancer diagnosis, vary by age, sex, and primary disease site. Lung, prostate, and breast are the most common sites for patients older than 25 years, while endocrine cancers and soft tissue sarcoma are the most common for those younger than 20 years [3]. Bone metastases increase the risk of serious complications, such as hypercalcemia, spinal cord compression, and skeletal-related events (SREs), including pathological fractures [4]. Bone metastases, as a sign of advanced disease, significantly affect the prognosis and lead to an increase in mortality and morbidity. However, nowadays, due to the progress in oncological treatments, the survival rates have significantly increased and range from a median of six months to five years, based on different factors, particularly the type of cancer, with liver, stomach, and lung displaying the worst 5-year survival rate, and breast and prostate the best [5].

In the face of notable progress in the oncological treatments, cancer-induced bone pain (CIBP) continue to be a major challenge for physicians. Most patients suffer from severe chronic pain, as a common symptom in bone metastatic disease, which may significantly affect their quality of life. Strong analgesics, such as opioids, radiotherapy, when applicable, and bisphosphonates are currently the mainstays of the management of bone metastatic disease [6]. However, reaching the optimal analgesic target is often hindered by the reduced tolerability of most analgesics, particularly opioids. Opioid-induced constipation (OIC) [7,8], analgesic tolerance, and opioid-induced hyperalgesia (OIH) [9] may limit their use. Moreover, recent concerns about the potential risk of respiratory depression, related to opioid misuse, have increased the need for alternative analgesic approaches [10]. Finally, the improved survival rate of these patients led to an increased awareness of the long-term use and related long-term adverse events of such analgesics, including the impairment of the endocrine system [11,12] and specific effects on bone metabolism, density, and healing [13,14]. In the last few years, atypical opioids, with a reduced mu-opioid receptor (MOR) activity, have been used as an alternative for cancer pain management [15,16]. Simultaneously, preclinical investigations have been conducted for identifying new mechanisms and innovative therapeutic targets for CIBP [17].

Targeting and regulating ion channels, which play a key role in modulating nervous system excitability, have been proposed as a potential therapeutic strategy for CIBP [18]. Among others, Transient Receptor Potential (TRP) ion channels have been shown to modulate pain perception in different conditions, characterized by nociceptive and neuropathic pain. These channels have been identified as essential molecules for detecting noxious stimuli, and for transducing thermal, mechanical, or chemical energy into electrical activity, namely into action potential in primary afferent fibres [19,20]. In the past few decades, these discoveries led to burgeoning research on their possible role in the pathogenesis of CIBP as well [21], which is still not fully understood. The aim of this narrative review is to provide an overview of the functional mechanisms of the TRP channel family, their involvement in the pathogenesis of CIBP, and their possible role for the analgesic management.

## 2. The TRP Channel Family

TRP channels are tetramers of subunits with six transmembrane-spanning segments (namely S1–S6), two cytoplasmic domains (COOH, known as C-terminal, and NH2, named N-terminal) with variable size, and a loop sequence forming a pore between segments S5-S6. The main differences between these channels are based on the structure of the intracellular cytoplasmic regions, with each specific family displaying specific residues. Such channels are ubiquitously expressed in several tissues in mammals, and are divided into subfamilies with different biophysical properties: TRPC (canonical), TRPA (Ankyrin), TRPM (melastatins), TRPML (mucolipins), TRPP (polycystins), TRPV (vanilloids), and TRPN (no mechanoreceptor potential C channels). Most TRP channels act as non-selective cation channels, mainly sensitive to calcium [21,22].

TRP channels can generally be activated by various mechanical, physical, chemical, and osmotic stimuli, and therefore, they are implicated in several physiological and pathological processes, ranging from taste and osmolarity perception, nociception, inflammatory conditions, and cancer. Actually, most TRP agonists and antagonists are not highly specific; not all TRP channels are modulated by the same molecules and stimuli, and there is cross-reactivity between these. Many natural compounds physiologically interact with the TRP channel family and induce a variety of sensations, such as warm, hot, cold, and pain. For instance, camphor, a terpenoid from the wood of the camphor laurel tree (*Cinnamomum camphora*), is a TRPV1 [23], TRPV3 [24], and TRPA1 agonist at low concentrations, as well as a TRPA1 antagonist at high concentrations, thus possibly mediating its analgesic effect [25]. Moreover, camphor activates TRPM8, thus resulting in sensitization to cooling [26]. Capsaicin (trans-8-methyl-N-vanillyl-6-nonenamide), the pungent compound in chilli peppers, activates TRPV1 [27], while TRPV2 and TRPV4 are not sensitive to it. Capsazepine, instead, is a capsaicin derivative that acts as a TRPV1 antagonist [28] and competitively inhibits the effects of capsaicin and resiniferatoxin. However, capsazepine may also interact with TRPM8 [29], and significantly attenuates cold allodynic response after chronic nerve injury [30]. Among natural compounds, ginger (gingerol) [31] and garlic (allicin) [32] activate both TRPA1 and TRPV1. Cannabinoids (such as cannabidiol), and endocannabinoids, such as arachidonoyl ethanolamine or anandamide, and 2-arachidonylglycerol (2-AG), alongside signalling molecules derived from arachidonic acid (AA) and polyunsaturated fatty acids, may modulate TRP channels [33]. Temperature stimuli may also activate specific channels (thermo-TRP). For instance, TRPM8 is activated by cool temperatures (≤25 °C), while TRPV1 is a sensor for higher temperatures (≥43 °C). TRPV4, TRPM2, TRPM3, TRPC5, and TRPA1 are also thermosensitive [34]. Moreover, products derived from oxidative stress and local acidosis, such as hydrogen peroxide (H_2_O_2_), nitric oxide (NO), and low pH (<6), may activate TRPV1, TRPA1, and TRPV4 [35]. 

## 3. TRP Ion Channels in the Pathogenesis of CIBP

Cancer-induced bone pain is a complex multifactorial phenomenon that is still not fully understood. When cancer cells reach and colonize the bone, they secrete Receptor Activator of Nuclear Factor κ B ligand (RANKL) and other pro-inflammatory factors, which promote the maturation of osteoclasts. Bone metastases grow simultaneously with the active bone resorption, leading to micro-fractures that activate nociceptors and induce CIBP [36]. The RANK pathway, among others, is currently a therapeutic target for CIBP for preventing skeletal-related complications [37].

TRP ion channels are implicated in bone metabolism, in different diseases, such as osteoporosis [38,39] and bone metastases. Several of these channels are implicated in osteoclast differentiation and were found in chondrocytes, in osteoblasts, and in mesenchymal stem cells [40]. TRP channels, by regulating intracellular calcium signalling, may interfere with tumour growth, proliferation, and spreading, especially in malignancies that often lead to bone metastases. Therefore, TRP channels may be a possible and appealing new target for managing bone metastases [41]. TRP channels have also been implicated in malignancies involving the bone marrow, such as in the hematological ones [42]. Particularly, recent evidence was brought out about a connection between various isoforms of TRP channels and the progression, spreading, bone destruction, and drug resistance in multiple myeloma (MM), which is known to be the second most commonly diagnosed hematologic malignancy. TRPV1 and TRPV2 are implicated with MM progression via the activation of osteoclasts, while TRPM7 promotes disease dissemination. The modulation of TRPVs 1 and 2 was correlated with different responsiveness to chemotherapy and chemotherapy-induced peripheral neuropathy (CIPN) [43].

At the same time, TRP channels are involved in the pathogenesis of bone pain. Some members of the TRP channel family have been shown to be involved, particularly in the peripheral mechanisms of CIBP [44,45]. It is well known that primary afferent neurons, namely A-δ and C fibres, which transmit the noxious stimuli from periphery to the dorsal root ganglion (DRG) neurons, establish neural connections with bones (periosteum, bone marrow, and mineralized bone) [46] and with tumour cells [47]. In particular, in recent decades, preclinical studies focused on the role of TRPV1 and TRPA1 in pain mechanisms. For instance, the up-regulation of tumour necrosis factor-α (TNF-α) and interleukin-6 (IL-6) in bone metastases may activate TRPA1 in the sensory neurons of bone cancer rats and contribute to CIBP [48]. The inflammatory mediators released in the metastatic niche and the acidic environment created by inflammation play a key role in activating TRP ion channels [49] and contribute to peripheral mechanisms of CIBP. The acidic tumour environment, caused by the release of protons (H^+^) by osteoclast activity, is a potent activator of TRP channels. Moreover, tumour acidosis increases the extracellular matrix remodelling and facilitate metastatic tumour cells to invade and grow into bone [50]. As protons are well-known TRPV1 activators, treatment with a blocker of proton secretion, such as bafilomycin A1, has been proven efficacious for CIBP relief [51]. The malignant infiltration of bone tissue is accompanied by an increase in neural density compared to healthy bone; nonetheless, the nerve profile density is lower in proximity of vascular structures and no correlation with tumour burden is appreciable, possibly implicating that nerve reorganization is mediated by paracrine and humoral factors, rather than direct contact with cancer cells [52].

Peripheral modifications are followed by alterations in excitability of sensory fibres in DRG [53], alongside an upregulation in excitatory channels, such as the purinergic adenosine triphosphate (ATP) receptors P2X3 [54] and P2X7 [55], overall resulting in heightened sensitivity and transmission through Aδ and C nerve fibres in the spinal cord [56]. Moreover, an imbalance in neurotransmitters in the central nervous system (CNS) was correlated with CIBP in animal models, ranging from a reduction in inhibitory [57] to an augmentation in excitatory transmissions [58,59]. Such modifications also take place in brain regions related to pain perception, such as the prefrontal and cingulate cortex, the dorsal hippocampus, the ventral tegmental area (VTA), and periaqueductal grey (PAG) [60,61,62]. Hence, the modulation of such neurotransmitters may be a therapeutic strategy against CIBP [63,64,65]. A firm correlation between nerve sprouting and pain perception is further supported by the fact that many molecules were found to cause neuroinflammatory responses and painful manifestations. For instance, TNF-α [66] and the nuclear factor kappa-light-chain-enhancer of activated B cells (NF-κB) [67] are activated in DRG neurons and microglia in animal models of cancer pain, leading to neuronal excitation. IL-6 [48] and interleukin-17 (IL-17) [68] induced neuropathic manifestations in animal models of bone cancer. Similar findings were observed for inflammatory lipid mediators, such as prostaglandin E2 (PGE2) [69], and chemokines, namely granulocyte–macrophage colony-stimulating factor (GM-CSF) [70] and chemokine (C-X-C motif) ligand 1/C-X-C motif chemokine receptor 2 (CXCL1/CXCR2), which were all correlated with the stimulation of pain receptors and sensory neurons and with the development of CIBP [71].

A role in CIBP is also plausible for neurotrophins, especially nerve growth factor (NGF) and its receptor tyrosine receptor kinase A (TrkA), which are implicated in peripheral nervous system inflammation and neuroinflammatory responses leading to long-term modifications of sensory neuronal function [72] and sensitization in CIBP [73]. Blocking the NGF/TrkA pathway attenuates the development of cancer-induced bone pain [74]. Tumours, indeed, display their own innervation, a well-known phenomenon called “neo-neurogenesis”; the more prominent the innervation, the more aggressive the cancer and the worse the prognosis [75]. In fact, cancer cells produce growth factors, namely NGF, which stimulate nerve sprouting, especially via the stimulation of new adrenergic nerve fibres and through the switch of sensory fibres into adrenergic ones [76,77,78]. NGF may also induce the expression of TRPV1 and it is implicated in cancer progression and CIBP [79].

**Figure 1:**
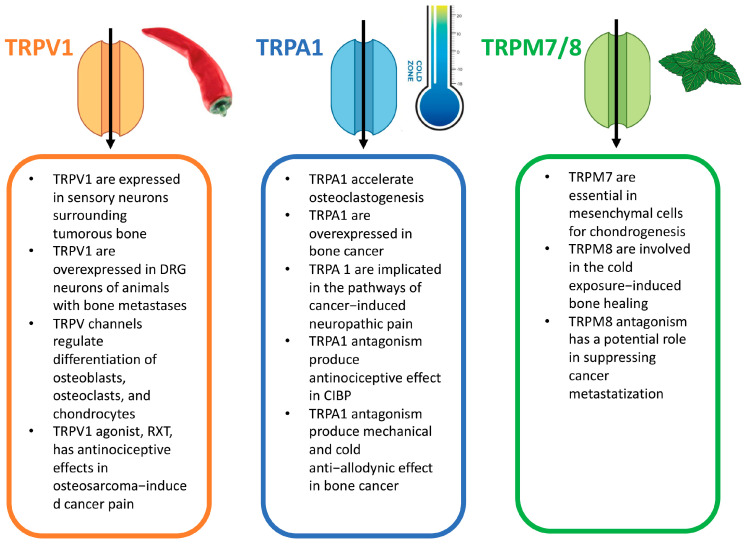
TRP channels involved in cancer-induced bone pain [21].

Some channels of the TRP family have been implicated in bone cancer, bone metastases, and CIBP, particularly some members of the TRPV, TRPA, and TRPM subfamilies, as shown in Figure 1 [21].

### TRP Channels and Neuro-Immune Modulation in Cancer-Induced Bone Pain

In the last few years, research focused on the role of TRP channels in the complex interplay between neurons and immune cells. TRP channels are indeed expressed in both these cells, with a possible role on carcinogenesis and metastases [80,81]. TRPV1 are the potential link between inflammation, cancer, and immunity. They are especially expressed on sensory neurons, which convey information from periphery to the CNS. These neurons produce and release pro-inflammatory neuropeptides, such as calcitonin gene-related peptide (CGRP) and substance P (SP), which trigger neuroinflammatory responses, via interaction with immune cells, the latter also expressing TRPV1 channels. Decreased TRPV1 expression was associated with increased risk of cervical squamous cell carcinoma [82], with poor clinical outcomes in renal cell carcinoma [83], and with an overall worse chance of survival. According to these findings, TRPV1 expression may have a prognostic value; it has been proven that TRPV1 have a positive correlation with the presence of immune-stimulatory cells, rather than immunosuppressive ones, in several cancer types including oral, ovarian cancer, and melanoma [84].

It has been supposed that the imbalance between the immune system and the CNS may cause serious consequences in cancerous conditions. For instance, chronic stress and inflammation may suppress protective immunological responses [85]. The nervous system itself may alter protective immunity against cancer [86] via multiple pathways; for example, the hypothalamic–pituitary–adrenal axis [87]. The activation of adrenergic sympathetic nerves promotes the formation of solid tumours [88] while the parasympathetic system exerts anti-tumoural and anti-metastatic effects. For instance, vagal visceral afferent nerve endings express TRPV1 [89]: their activation decreased breast cancer metastasis [90,91] and reduced pro-inflammatory cytokines [92], all while upregulating the release of SP [93].

TRPV1 may modulate pain pathway also through central effects. They are expressed in the brain (hypothalamus, hippocampus, and midbrain) and in microglia and astrocytes, which, as resident immune cells of the CNS, are the main actors of neuroinflammation [80].

**Figure 2:**
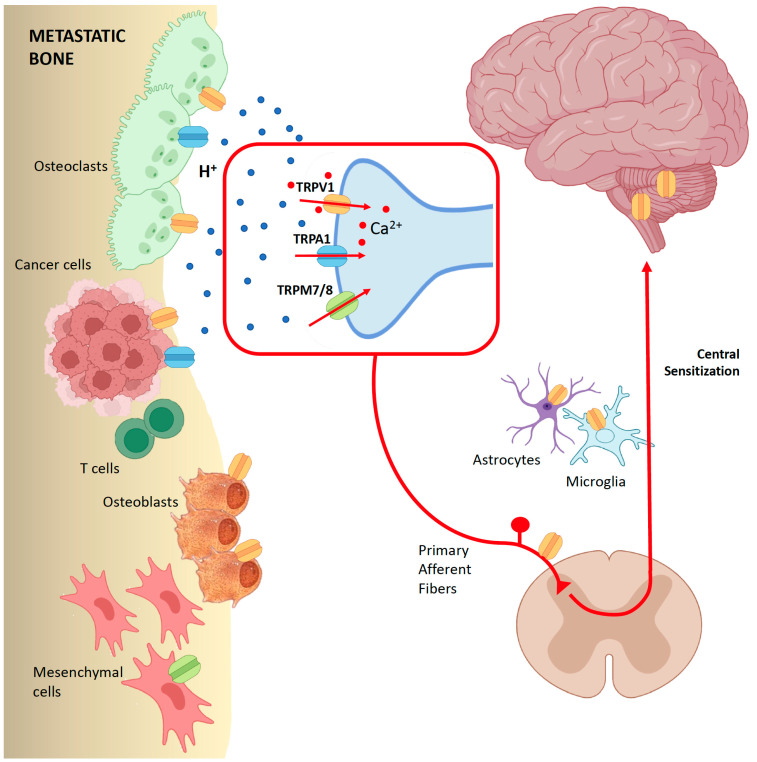
TRP channels in metastatic bone disease: modulation of sensory neurons and immune cells. Cancer cells stimulate osteoclastic activity in the metastatic bone. Osteoclasts release protons (H^+^) and reduce the pH of bone environment, which activates TRP channels. In particular, TRPV1 channels are expressed in the peripheral terminal of primary afferent fibres. TRPV1 channels are also expressed in the central nervous system, not only in dorsal root ganglion neurons but also in glial cells and astrocytes, which contribute to central sensitization and neuro-immune balance in neuropathic pain. TRPV1 also act directly on bone cells, by modulating the differentiation of osteoclasts and osteoblasts; therefore, they are a possible therapeutic target for bone diseases such as osteoporosis and skeletal metastases. Finally, TRPV1-positive sensory neurons may release substances that affect tumour-infiltrating immune cells. TRPV1 are involved in the interplay between immune cells, such as T cells, and cancer cells; therefore, their role in carcinogenesis could be the result of their effect on the immune system. TRPA1 are expressed both in osteoclasts and in cancer cells. TRPA1 are also implicated in pain pathways in bone cancer. TRPM 7/8 regulate the activity of mesenchymal cells and play a role in cancer spreading and metastases.

Finally, TRPV1 are expressed also on T cells and natural killer (NK) cells [94]. TRPV1 agonists increase the calcium (Ca^2+^) flux and the intracellular concentration of calcium in T cells, leading to their apoptosis, while the effects on NK cells are still unknown [80]. Sensory neurons, through the release of CGRP, may impair the cytotoxic potential of tumour-infiltrating CD8+ T cells, leading to their exhaustion [95]. The repeated activation of TRPV1-positive sensory neurons reduces intra-tumour CD4+ T cells and possibly promotes tumour growth [96]. All these findings support further investigation for elucidating the role of the TRP channel family in the complex relationship between cancer cells, immunity, and the nervous system (Figure 2).

## 4. TRPV Channels

The TRPV subfamily consists of six members, further classified into four groups, namely TRPV1/TRPV2, TRPV3, TRPV4, and TRPV5/TRPV6. These are hetero- or –homo-tetrameres expressed on plasma membranes (PMs), with each subunit usually including a TRP box and three to five N-terminal ankyrin repeats at the C terminal. While TRPV5 and TRPV6 are highly selective Ca^2+^ channels with strict regulation by intracellular calcium concentrations ([Ca^2+^]i), TRPV1, TRPV2, TRPV3, and TRPV4 are only moderately permeable to Ca^2+^ [20,97]. Channel activation leads to the entrance of Ca^2+^ through the PM and its release from endoplasmic reticulum [98] and mitochondria storage; thus, the activation of Ca^2+^-dependent signalling pathways, namely phosphatidylinositol 3-kinase/protein kinase B (PI3K/PKB) [99], mitogen-activated protein kinase/extracellular signal regulated kinase (MAPK/ERK), and calmodulin, also activate ERK [100]. Such pathways are well known to be involved in tumour proliferation, survival, and progression [101,102,103].

TRPV1 was first described in 1997, when it was cloned from vertebrates, and it was named the “capsaicin receptor”, as it was identified as the only channel to be sensitive to vanilloid capsaicin. TRPV1 has a molecular size of 95 kiloDalton (KD) and consists of 838 amino acids [104] and is expressed in both neuronal and non-neuronal cells. In neuronal cells, after being synthetized in the nucleus, TRPV1 is transferred to the Golgi apparatus and, from here, to the synaptic membrane through vesicles moving along the microtubules, whilst forming a complex with transport and linker proteins, namely kinesin-13B (KIF13B). This complex dissociates once it has reached the PM, thus expressing TRPV1 receptors on it [105]. TRPV1 is particularly present in peripheral small unmyelinated C-fibres and in pain-sensitive neurons in the DRG [106]. It is also present in several brain regions, namely periaqueductal grey, locus coeruleus, substantia nigra, midbrain, and hypothalamus [107], as well as in trigeminal afferents [108]. Normally, TRPV1 is mainly expressed in small and medium DRG neurons. However, in several painful conditions, ranging from diabetes to neuropathic pain and cancer pain, a change in TRPV1 localization and expression is possible in distinct subpopulations of DRG neurons. Particularly, in cancer pain models, TRPV1 is expressed in larger DRG neurons than usual [45]. Among DRG neurons of mice with osteosarcoma femur implantation, TRPV1 were shown to be co-localized with CGRP, a marker of peptidergic neurons, and the neurofilament 200 kD (NF200), a marker of neurons with myelinated fibres, but not with isolectin B4 (IB4), which is a marker of nonpeptidergic unmyelinated neuron [106]. Nonetheless, after implantation of mammary rat metastasis tumour (MRMT-1) tumour cells into the tibial canal in rats, Zheng et al. found that cancer-induced thermal and mechanical hyperalgesia were correlated with the amplified excitability of small-sized TRPV1- and IB4-positive DRG neurons [53].

TRPV1 acts as polymodal integrator of painful stimuli, since it is also found in the skin [109], muscles [110], internal organs (e.g., epithelium of the bladder [111]), cardiovascular structures [112], synoviocytes [113], osteoblasts, and osteoclasts, with a role in regulating their differentiation and function [114]. TRPV1 is activated by noxious heat (≥43 °C) and irritant compounds, such as the aforementioned capsaicin, as well as resiniferatoxin (RTX), which is found in the latex of the Euphorbia resinifera, a cactus-like plant, piperine (present in black pepper), eugenol (found in cloves), allicin (found in garlic), gingerol, and zingerone (from ginger), alongside known venoms from spiders, scorpions, jellyfish, and others [115]. TRPV1 is also activated by both acidic and basic deviations from homeostatic pH [116]; nonetheless, low pH (<5.9) is its main regulator [117], which is typical in the acidic microenvironment driven by bone cancer [118]. TRPV1 is also activated by inflammatory molecules, namely endovanilloids [119], serotonin (5-HT) [120], histamine [121], formaldehyde [122,123], lipid metabolites [124], prostaglandins (PGs) [125], bradykinin (BK) [126], ATP [127], TNF-α [128], granulocyte colony-stimulating factor (G-CSF) [129], high-mobility-group box 1 (HMGB1) [130], parathyroid hormone-related peptide (PTHrP) [131], transforming growth factor-β1 (TGF-β1) [132], NGF [133], interleukin-17A (IL-17A) [134], IL-6 [135], and others, resulting in painful hypersensitivity.

Particularly, among other inflammatory molecules, insulin-like growth factor-1 (IGF-1) was increased in rat tibia bone marrow after MRMT-1 cells inoculation. IGF-1 is normally implicated in osseous metabolism, promoting osteoblast differentiation, mitosis, and bone construction, with neurotrophic effects after nerve injury, which inevitably occurs during metastasization to the bone when malignant cells disrupt the bone cortex, allowing IGF-1 to reach the nerve endings expressed here. In this scenario, IGF-1 is thought to contribute to pain pathogenesis in cancer; the stimulation of IGF-1-receptor produces mechanical allodynia and thermal hyperalgesia. Accordingly, incubation with IGF-1 was shown to upregulate TRPV1 expression and TRPV1-derived current density, with enhanced sensitivity to capsaicin, and increase in TRPV1 co-localized with IGF-1 receptor in small DRG neurons. As a consequence, a 3-day-long intraperitoneal administration of picropodophyllotoxin (PPP), an IGF-1 receptor inhibitor, reversed tumour-induced thermal hyperalgesia and mechanical allodynia. Moreover, PPP was found to inhibit tumour cell growth via inhibiting IGF-1 receptor (IGF-1R) phosphorylation and starting PKB-mediated pathways [136]. Anyway, a role for IGF-1 in pain regulation via TRPV1 modulation is even more plausible when considering the protective role of insulin in diabetic painful neuropathy and the TRPV1 overexpression found in case of insulin resistance [137]. Accordingly, metformin was found to have analgesic effects in visceral, inflammatory, and neuropathic pain via the inhibition of TRPV1 and other acid-sensing channels [138].

TRPV1 activators ultimately act via G protein-coupled receptors (GPCRs), namely protease-activated receptor-2 (PAR2), that generate second messengers such as inositol triphosphate (IP3), diacylglycerol (DAG), and phosphatidylinositol-4,5-biphosphate (PIP2). Consequently, effectors such as protein kinase C (PKC) [124], protein kinase A (PKA) [139,140,141], Ca^2+^/calmodulin-dependent protein kinase II (CaMKII) [142], and Janus kinase (JAK)/PI3K [135] are activated, starting signals for pain regulation and enhancement. Studies found that the inhibition of such receptors and effectors attenuates TRPV1-induced hyperalgesia in cancer pain models, and may even ameliorate opioid resistance [143], which is quite common in cancer pain, because peripheral nerve injury, occurring during tumour cells invasion in the bone, may alter the expression of MOR and signalling proteins in the spinal cord [144,145]. Moreover, Ca^2+^ influx intensifies when TRPV1 is activated, thus starting membrane depolarization and allowing for the further activation of voltage-gated sodium channels and the generation of action potentials, eventually increasing nociception [104].

TRPV1 was first assessed in bone cancer pain in a 2005 study, via the injection of osteolytic sarcoma cells in mouse femurs. It was found that most sensory neurons in tumorous bone expressed TRPV1 and subcutaneous administration of its antagonist JNJ-17203212 reduced pain-induced behaviours [146]. Further studies showed that TRPV1 was overexpressed in DRG neurons in animals after an injection of the breast cancer cells Walker 256 into tibial and femur bone cavities, with injected rats demonstrating mechanical allodynia, increased spontaneous flinching, and guarding, as proof of spontaneous pain and decreased limb use [147].

With regard to the other components of the TRP family, TRPV2 [148] and TRPV4 [149] are also expressed in DRG neurons, while TRPV3 is mainly found in the brain [150], as well as keratinocytes [151], cells surrounding hair follicles [152], oral mucosa [153], and so on. TRPV4 is also expressed in blood vessels [154], keratinocytes [155], skeletal muscle cells [156], pancreas [157], and in various bone cell types [158], including mesenchymal stem cells [159]. TRPV channels are generally believed to regulate renal calcium reabsorption [160], skeletal homeostasis [161], and the differentiation of osteoblasts, osteoclasts, and chondrocytes [38,162,163].

### 4.1. TRPV Modulation in Cancer-Induced Bone Pain

The use of TRPV1 antagonists and the knockdown of TRPV1 protein resulted in reduced sensitivity to nociception in preclinical pain models, for instance in neuropathic, osteoarthritic, postoperative pain, and cancer pain, with special regard to CIBP, with variability in their analgesic effects, possibly due to differences in their pharmacological properties. Nonetheless, their analgesic effect has not been thoroughly evaluated in patients with cancer pain. TRPV1 antagonists have been associated with a series of adverse effects, such as hyperthermia or hypothermia, and the altered perception of noxious heat, with risk of burn damage; this caused the premature suspension of some clinical trials and spurred the urgency to find selective and more tolerable compounds [21]. Many TRPV1 modulators have also been evaluated as cancer therapies, given their effect on cancer cell proliferation through excessive Ca^2+^ influx, which is knowingly cytotoxic [164]. The genetic deletion of TRPV1 reduced nocifensive behaviour and hyperalgesia in mice [165], alongside a reduction in sensory nerve excitation in the DRG and spinal dorsal horns [44]. Suitably, TRPV1 knockdown through an adeno-associated virus (AAV)-mediated short-interfering RNA (siRNA) reduced mechanical allodynia and thermal hyperalgesia in a rat model of bone cancer pain, with concomitant under-expression of neuroinflammatory mediators in the animals’ spinal cords [166]. A siRNA against TRPV1, called Tivanisiran (SYL1001), developed by Sylentis, was tested up to Phase 3 in patients with dry eye disease, reducing ocular pain [167].

#### 4.1.1. Resiniferatoxin (RTX)

RTX is an ultra-potent capsaicin analogue, with an approximately 1000-fold higher potency, found in the latex of a cactus-like plant, Euphorbia resinífera. Hence, it acts as a TRPV1 agonist by either capsaicin or proton activation, leading to calcium influx and channel desensitization, as well as the defunctionalization of the pain fibres, with consequent analgesic effects in several painful conditions, namely diabetic neuropathy, chronic phantom pain, and cancer pain. Hence, it should only be injected in specific and limited sites to avoid systemic effects. In the future, RTX will be available as intra-articular injection for pain relief in knee osteoarthritis [168]. Its role in cancer pain management is currently being investigated. In preclinical cancer pain models, RTX was found to have antinociceptive effects in dogs [169] and mice [170] with osteosarcoma-induced cancer pain, when administrated intrathecally and subcutaneously, respectively. Moreover, the intrathecal administration of RTX ameliorated intractable pain in patients with advanced cancer [171]. At present, the efficacy of RTX against cancer pain has been evaluated in a restricted number of clinical trials [172]. The first-ever trial (NCT00804154) assessed the analgesic properties of intrathecal RTX in patients with metastatic bone disease [173]. In one of these, intrathecal RTX was tested in patients with advanced cancer and refractory pain (Phase 1b, recruiting, identifier NCT00804154, National Institute of Dental and Craniofacial Research—NIDCR) (Clinical Trials, NCT00804154, 2008). In another trial (Phase 1b, not yet recruiting, identifier NCT02522611, National Institute of Neurological Disorders and Stroke—NINDS), RTX was administered via the periganglionic route to study its effects on CIPB in refractory patients (Clinical Trials, NCT02522611, 2015). In a third trial, RTX was administrated epidurally in patients with advanced cancer (Phase 1b, ongoing, identifier NCT03226574, Sorrento Therapeutics) (Clinical Trials, NCT03226574, 2017); a short-term analysis of this multicenter study, performing open-label dose-escalation, suggested positive results. In particular, in 14 patients receiving lower doses of RTX (0.4, 1, 2, and 4 μg) there was no sensible reduction in cancer pain, while 3 patients who were administered higher doses (8 and 15 μg) via the epidural route had a decrease in pain, with post-procedure-related pain being the most common side effect (50%) but with total resolution in a two-day period of time. Hence, higher doses of RTX (up to 25 μg) were studied in an additional cohort in Phase 3 studies, with 17 patients being administered 0.4–25 μg of RTX with no considerable side effects and with dose-dependent analgesia [21].

#### 4.1.2. JNJ-17203212

To contrast to RTX, JNJ-17203212 is a potent selective antagonist of TRPV1 activation by either capsaicin or protons [146]. In mice treated with an injection of osteolytic NCTC 2472 sarcoma cells, JNJ-17203212 reduced both movement-evoked and ongoing nocifensive behaviours at different times throughout cancer progression, with no effect on tumour growth in the sarcoma-bearing femur. Moreover, chronic treatment with JNJ-17203212 blocked c-Fos expression in the spinal cord, which is usually correlated with pain perception [165].

#### 4.1.3. SB366791 [N-(3-Methoxyphenyl)-4-chlorocinnamide]

SB366791 partially inhibits capsaicin-induced acute nociception when administrated intraperitoneally at a 0.5 mg/Kg dosage, while it is not a TRPV1 antagonist after proton-induced activation. In C3H/HeJ mice injected with osteolytic sarcoma cells, SB366791 potentiated morphine-induced analgesia. This could open the way for future combination treatments for cancer pain [144].

#### 4.1.4. 5-Iodoresiniferatoxin

5-iodoresiniferatoxin (I-RTX) is a strong TRPV1 antagonist, derived from RTX [174]. In mice inoculated with NCTC 2472 cells, an intraperitoneal injection of I-RTX had antinociceptive effects and caused an increase in TRPV1 expression in L2/3/4 small, medium, and large DRGs and peripheral axons ipsilateral to the sarcoma cell injection [106].

#### 4.1.5. ABT-102

Spontaneous pain induced by NCTC 2472 sarcoma cell injection was reduced by oral administration of TRPV1 antagonist ABT-102 [175].

#### 4.1.6. Capsazepine (CPZ)

A subcutaneous injection of squamous cell carcinoma (SCC-7) [176], SCC-158 cells [177], or osteosarcoma NCTC 2472 cells [170] in animals caused heat hyperalgesia and higher TRPV1 expression in DRG, which were reduced via the administration of capsazepine (CPZ). CPZ can modulate the cystine/glutamate antiporter, which was linked to CIBP induction via excessive glutamate secretion from distal breast cancer metastases. The administration of CPZ delayed the onset and reversed CIBP-induced nociceptive behaviours after intrafemoral MDA-MB-231 breast tumour cells [178].

#### 4.1.7. QX-314

QX-314 is a quaternary lidocaine derivative with a positive charge that would hinder its ability to cross neuronal membranes. QX-314 has biphasic effects on TRPV1 channels; in fact, it inhibits capsaicin-evoked TRPV1 currents at lower (micromolar) concentrations, while it activates TRPV1 channels at higher (millimolar) concentrations in vitro [179]. Moreover, it was found to directly activate and permeate the human isoform of TRPV1, with consequent inhibition of sodium channels [180]. QX-314 has a probable role in inhibiting pain, particularly thermal hyperalgesia and flinching behaviour, transmitted by TRPV1-expressing afferents, while maintaining motor function and proprioception in animal models. It inhibits ectopic discharges from DRG neurons after nerve injury and the increased activity of spinal dorsal horn neurons after skin incision [181]. These findings hint at a possible role for QX-314 in the field of pain management in humans via the modulation of TRPV1 channels.

#### 4.1.8. Quercetin

Quercetin inhibits expressions of several molecules within the PAR2/TRPV1 pathway, namely PAR2, TRPV1, PKA, and PKC-γ in the DRG neurons in rats with bone cancer pain, as well as inflammatory mediators and cells, such as TNF-α, IL-1β, and macrophages, thus hindering the peripheral and central sensitization of bone cancer pain. Normally, when activated, PAR2 can either be coupled with GAS gene, which directly activates PKA, or with the GaQll gene to activate phospholipase C (PLC), to eventually activate PKC: activated PKC and PKA phosphorylate and activate TRPV1 [182].

#### 4.1.9. Acetaminophen

Studies found that the antinociceptive effects of acetaminophen are lost in TRPV1 knockout (KO) mice. Acetaminophen metabolites N-arachidonoylphenolamine (AM404) and N-acetyl-4-benzoquinoneimine (NAPQI) can bind to the vanilloid binding site and activate TRPV1 in DRG [183].

#### 4.1.10. Xiaozheng Zhitong Paste (XZP)

Herbal analgesic Xiaozheng Zhitong Paste (XZP) is used in traditional Chinese medicine for cancer pain analgesia. In breast cancer-induced bone pain through the inoculation of Walker 256 cells into Wistar rats, topical XZP mitigated bone cancer-related nociceptive behaviour by inhibiting the PAR2/TRPV1 signalling via a reduction in PKA, PKC-γ, PAR2, and TRPV1 levels, alongside trypsin, IL-1β, and TNF-α serum levels. It also contained bone damage, with positive effects on bone mineral density (BMD) and bone mineral content (BMC) [184].

#### 4.1.11. Quetiapine

Intraperitoneal administration of quetiapine improved the paw withdrawal pressure threshold, as a sign of reduced nociception, and was correlated with lower mRNA levels of TRPV1 and TRPV4 in C3H/HeN mice with cancer pain [185].

#### 4.1.12. Arachidonyl-2-chloroethylamide

Kawamata et al. found that spinal cannabinoid receptor 1 (CB1) activation by the CB1 agonist arachidonyl-2-chloroethylamide reduced bone cancer-related spontaneous and movement-evoked pain with a dose-dependent pattern [186].

## 5. TRPA Channels

TRPA1 is actually the only member of the TRPA subfamily; it is a non-selective Ca^2+^ permeable cation channel, displaying a voltage sensor and a calcium-binding domain in the C-terminal, 16 ankyrin repeat sequences in the N-terminal domain, and a putative selectivity filter at the entrance of the pore [187]. TRPA1 is expressed in various neuronal and non-neuronal cytotypes, colocalizing with TRPV1: in fact, it can be found in the brain [188], in DRG, and trigeminal ganglion (TG) neurons [189], as well as other tissues, such as in the small intestines [190], lungs [191], bladder [192], inner ear [193], cardiovascular structures [194], skeletal muscles [195], odontoblasts [196], and others. It was found to be expressed in malignant tissues, such as oral squamous cell carcinoma [197], nasopharyngeal carcinoma [198], breast carcinoma [199], pancreatic adenocarcinoma [200], colorectal cancer [201], and prostate cancer [202]. A higher expression of TRPA1 was correlated with decreased migration of tumour cells and improved survival in osteosarcoma [203]. TRPA1 is also highly represented in C and Aδ nerve fibres and may be activated by mechanical stimuli and cold temperatures (<18 °C), while other sources claim it to be sensitive to temperatures ranging from 17 to 40 °C [204]. Moreover, it can be activated by chemical irritants, exogenous compounds such as allyl isothiocyanate (AITC, mustard oil), allicin, and cinnamaldehyde [205], as well as pH changes and inflammatory molecules and products (e.g., H_2_O_2_ [206] and PGs [207], with possible cross-sensitization and/or desensitization with TRPV1 [208]. In fact, in a model of pancreatic pain, pancreatic inflammation augmented both TRPV1 and TRPA1 expression and the excitability of sensory neurons, which were reduced via the administration of TRPV1/A1 antagonists, with additive effect [209]. Furthermore, similarly to TRPV1, acetaminophen and its metabolite NAPQI sensitize and activate TRPA1 by interacting with intracellular cysteine residues, leading to a reduction in voltage-gated calcium and sodium currents in DRG neurons, with antinociceptive results, the latter being lost in TRPA1 KO mice [183].

### TRPA1 Modulation in Cancer-Induced Bone Pain

TRPA1 are implicated in bone-remodelling diseases, such as osteoporosis, as they accelerate osteoclastogenesis. Their expression gradually increases in the osteoclast differentiation process [210]. TRPA1 are also of significant interest for their role in metastasis and overexpression in bone cancer, such as osteosarcoma [211]. They could be a promising strategy for pain relief in CIBP in the future. TRPA1 have been shown to mediate mechanical allodynia and thermal hyperalgesia in a rat model of CIBP [212]. TRPA1 are upregulated in rats with bone cancer pain, where they have been implicated in the pathways leading to neuropathic pain [48]. TRPA1-deficient mice did not display mechanical and cold allodynia and thigmotaxis behaviour after the injection of B16-F10 murine melanoma cells into the plantar region of the right hind paw, with no effect on paw thickness as an expression of cancer growth [213]. H_2_O_2_ levels and Nicotinamide Adenine Dinucleotide Phosphate Hydrogen (NADPH) oxidase activity were increased in injected animals; therefore, antioxidant α-lipoic acid attenuated all symptoms in TRPA1+ animals, except for cold allodynia [214].

HC-030031, a TRPA1 antagonist, has been shown to produce an antinociceptive effect in mice injected with breast cancer cells. Repeated administration of a TRPA1 antagonist has been shown to produce mechanical and cold anti-allodynic effect. No effects were recorded on the tumour growth [215]. These findings suggest TRPA1 receptors as a potential target for new analgesic approaches to BICP.

## 6. TRPM Channels

The TRPM subfamily enlists eight different channels, namely TRPM1 to TRPM8. All of them are made of four domains, named melastatin homology regions (MHR1-MHR4), with one pre-S1 domain in their N-terminus. The C-terminus contains the TRP box with a highly conserved amino acid sequence, which is pivotal for channel stability within the plasma membrane, and is followed by a coiled–coil domain for the assembly of tetrameric complexes [216]. The transcriptional regulation of TRPM genes allows for diversity in channel structure: particularly, added domains with enzymatic function may be present, and there is heterogeneity in activation mechanisms, as well as regulators. Some TRPMs, especially TRPM2, TRPM3, TRPM4, TRPM5, and TRPM8, are sensitive to a wide range of temperatures; hence, they are called thermoTRPs [217].

Some of these channels, particularly TRPM3 and TRPM8, are possible targets for pain control: in fact, TRPM3 is blocked by non-steroid anti-inflammatory drugs (NSAIDs) and is regulated by G-proteins, similarly to TRPM8; hence, they both may be blocked after opioid receptor activation [218]. Trials for specific antagonists of TRPM3 and TRPM8 are currently ongoing, while menthol, as a TRPM8 inhibitor, is the only one currently approved for clinical use in patches [219].

Some isoforms of TRPM channels were found to have a role in cancer metastasization, besides primitive cancer growth and local invasion. TRPM2 knockdown was correlated with reduced tumour proliferation and the deregulation of metastatic markers in gastric adenocarcinoma (AGS), non-small lung cancer (NSCLC) and cell lines [220]. The overexpression of TRPM5 led to an increased degree of pH-induced matrix metalloproteinase-9 (MMP-9) and lung metastasis in mice injected with B16-BL6 cells, with an evident reduction after administration of TRPM5-inhibitor triphenylphosphine oxide (TPPO) [221]. TRPM7 was overexpressed in NSCLC cell line A549 after stimulation with epidermal growth factor (EGF), with consequent increase in cell migration: both TRPM7-KO and the use of Waixenicin A counteracted the elevation in TRPM7 levels and had anti-cancer stem cell (CSC) effects [222]. A role for TRPM7 is also possible for the dissemination of MM cells, probably via TRPM7-mediated Ca^2+^ influx and the consequent activation of Integrin Subunit Alpha 4 (ITGA4) and Integrin Subunit Beta 7 (ITGB7) [223]. TRPM8 is expressed in C and Aδ nerve fibres in DRG and TG neurons, as well as on osteoblasts, even though its role in the latter is not quite clear. TRPM8 is activated by both cold temperatures (<15 °C) and cooling compounds, namely peppermint oil, icilin, and menthol. On the other hand, whether TRPM8 is activated by acid is still unclear. TRPM8 was first described in prostate cancer and cloned as a molecule with high homology to a TRP-like channel; later on, its presence in several cancers, also including lung, gastric, liver, ovarian, melanoma, and breast, was assessed [224,225]. TRPM8 seems to have a role in cancer cell proliferation and metastasization, since the incubation of osteosarcoma cell lines with AMTB, a TRPM8 antagonist, suppressed such processes and induced apoptosis through the regulation of TGF-β pathways [226]. Given TRPM8 is overexpressed in oral squamous cell carcinoma (SCC), its antagonist RQ-00203078 was used to prove a reduction in the invasion and migration capability of SCC cancer cells, via a reduction on calcium influx [227]. Similarly, TRPM8 is overexpressed in malignant prostate tissues, with androgen dependency and direct interactions with androgen receptor (AR), especially in hormone refractory cancers. TRPM8 inhibitors AMT, JNJ41876666, and BCTC reduced proliferation rates in malignant prostate cell lines, namely DU145, LNCaP, and PC3 [228]. In the latter two, TRPM8 depletion was also correlated with enhanced chemosensitivity towards epirubicin via the phosphorylation of JNK and p38 proteins [229]. Conversely, the TRPM8 agonists menthol and WS12 reduced cancer cell proliferation and migration ability [230].

### TRPM Modulation in Cancer-Induced Bone Pain

To the best of our knowledge, no data are available about the role of TRPM channels in CIBP. TRPM7 in mesenchymal cells have been shown to be essential in regulating chondrogenesis. They play a key role, as cation channels for Ca^2+^ and magnesium (Mg^2+^), for bone development [231]. TRPM8 are involved in cooling-promoted bone healing: cold exposure induced vasoconstriction and an increase in TRPM8 within cortical defects, associated with increased vascular endothelial growth factor (VEGF) and angiogenesis, which promotes bone regeneration [232]. TRPM8 antagonism has also a potential role in suppressing cancer metastasization [226]. A role for TRPM channels in bone formation and metabolism is plausible [231,233,234,235,236]; accordingly, the upregulation of TRPM8 [237] and TRPM3 [238] was associated with non-malignant chronic low back pain.

## 7. Conclusions

CIBP is still undermanaged and presents a number of unmet needs. Traditional analgesics, such as opioids, are burden by a number of side effects, which may limit their use and occasionally may represent a severe risk for frail cancer patients. On the other hand, due to the enormous progress in cancer treatments, the relatively long-life expectancy of patients with metastatic disease supports research on innovative analgesic strategies that may overcome the long-term side effects of opioids. TRP cation channels are involved in the regulation of various characteristics of cancer cells. Their role in CIBP is still poorly understood, but is currently the subject of active investigations. Being a large and variegate family, with interference not only on nerve fibres, but also directly on cancer cells, their role in cancer pain management is an appealing field of research. Future studies in this field may identify some types of TRP channels as possible markers or therapeutic targets for preventing progression and treating bone metastatic disease in patients with cancer.

## Abbreviations

The following abbreviations are used in this manuscript:

2-AG	2-arachidonylglycerol
5-HT	Serotonin
AA	Arachidonic acid
AAV	Adeno-associated virus
AGS	Gastric adenocarcinoma
AITC	Allyl isothiocyanate
AM404	N-arachidonoylphenolamine
AR	Androgen receptor
ATP	Adenosine triphosphate
BK	Bradykinin
BMC	Bone mineral content
BMD	Bone mineral density
Ca^2+^	Calcium
[Ca^2+^]i	Intracellular calcium concentrations
CaMKII	Ca^2+^/calmodulin-dependent protein kinase II
CB1	Cannabinoid receptor 1
CGRP	Calcitonin gene-related peptide
CIBP	Cancer-induced bone pain
CIPN	Chemotherapy induced peripheral neuropathy
CNS	Central nervous system
CPZ	Capsazepine
CSC	Cancer stem cell
CXCL1	Chemokine (C-X-C motif) ligand 1
CXCR2	C-X-C motif chemokine receptor 2
DAG	Diacylglycerol
DRG	Dorsal root ganglion
EGF	Epidermal growth factor
G-CSF	Granulocyte colony-stimulating factor
GM-CSF	Granulocyte–macrophage colony-stimulating factor
GPCRs	G protein-coupled receptors
H_2_O_2_	Hydrogen peroxide
HMGB1	High-mobility-group box 1
IB4	Isolectin B4
IGF-1	Insulin-like growth factor-1
IGF-1R	Insulin-like growth factor-1 receptor
IL-17	Interleukin-17
IL-17A	Interleukin-17A
IL-6	Interleukin-6
IP3	Inositol triphosphate
I-RTX	5-iodoresiniferatoxin
ITGA4	Integrin Subunit Alpha 4
ITGB7	Integrin Subunit Beta 7
JAK	Janus kinase
KD	KiloDalton
KIF13B	Kinesin-13B
KO	Knockout
MAPK/ERK	Mitogen-activated protein kinase/extracellular signal regulated kinase
Mg^2+^	Magnesium
MM	Multiple myeloma
MMP-9	Matrix metalloproteinase-9
MOR	Mu-opioid receptor
MRMT-1	Mammary rat metastasis tumour
NADPH	Nicotinamide Adenine Dinucleotide Phosphate Hydrogen
NAPQI	N-acetyl-4-benzoquinoneimine
NF200	Neurofilament 200 kD
NF-κB	Nuclear factor kappa-light-chain-enhancer of activated B cells
NGF	Nerve growth factor
NK	Natural killer
NO	Nitric oxide
NSAIDs	Non-steroid anti-inflammatory drugs
NSCLC	Non-small lung cancer
OIC	Opioid-induced constipation
OIH	Opioid-induced hyperalgesia
PAG	Periaqueductal grey
PAR2	Protease-activated receptor-2
PD-L1	Programmed death ligand 1
PGE2	Prostaglandin E2
PGs	Prostaglandins
PI3K/PKB	Phosphatidylinositol 3-kinase/protein kinase B
PIP2	Phosphatidylinositol-4,5-biphosphate
PKA	Protein kinase A
PKC	Protein kinase C
PLC	Phospholipase C
PM	Plasma membrane
PPP	Picropodophyllotoxin
PTHrP	Parathyroid hormone-related peptide
RANK	Receptor Activator of Nuclear Factor κ B
RANKL	Receptor Activator of Nuclear Factor κ B ligand
RTX	Resiniferatoxin
SCC	Squamous cell carcinoma
siRNA	Short-interfering RNA
SP	Substance P
SREs	Skeletal-related events
TG	Trigeminal ganglion
TGF-β1	Transforming growth factor-β1
TNF-α	Tumour necrosis factor-α
TPPO	Triphenylphosphine oxide
TrkA	Tyrosine receptor kinases A
TRP	Transient Receptor Potential
TRPA	Transient Receptor Potential Ankyrin
TRPC	Transient Receptor Potential canonical
TRPM	Transient Receptor Potential melastatins
TRPML	Transient Receptor Potential mucolipins
TRPN	Transient Receptor Potential no mechanoreceptor potential C channels
TRPP	Transient Receptor Potential polycystins
TRPV	Transient Receptor Potential vanilloids
VEGF	Vascular endothelial growth factor
VTA	Ventral tegmental area
XZP	Xiaozheng Zhitong Paste
